# Diversity and genetic structure analysis of three Amazonian Amerindian populations of Colombia.

**Published:** 2012-06-30

**Authors:** Yamid Braga, Leonardo Arias B, Guillermo Barreto

**Affiliations:** a Universidad del Valle. Sección de Genética, Departamento de Biología, Facultad de Ciencias Naturales y Exactas. Colombia Email: guillermo.barreto@correounivalle.edu.co

**Keywords:** Amazonia, East Tucanos, Guahiba, Indians of South America, microsatellite repeats, genetic variation, Guaviare, Vaupés

## Abstract

**Introduction:**

In the departments of the Vaupés and Guaviare, in southeastern Colombia, in a transitional area between Amazonia and the eastern plains, inhabit indigenous groups belonging to the Tukanoan (East) and Guahiban linguistic families. Although some studies have dealt with the culture and the cosmology description of these groups, little research has been done on the biological diversity and genetic relationships of such groups.

**Objective:**

To estimate the diversity, the structure, and the genetic relationships of one Guahiban and two Tukanoan groups of the Colombian Amazonian region.

**Methods:**

Samples were collected (n = 106) from unrelated individuals belonging to the Vaupés native indigenous communities. The DNA was extracted and nine autosomal microsatellites were typed. Several measures of diversity, FST, pairwise FST, and population differentiation between groups were calculated. Finally, it was estimated the genetic distances of the groups studied in relation with other Amazonian, Andean and Central American indigenous people.

**Results:**

1. The genetic diversity found stands within the range of other Amazonian populations, whereas compared to the mestizo and afro-descendant Colombian populations, such diversity showed to be lower. 2. The structure and population differentiation tests showed two clusters; one consisting of the Vaupés Tukanoan and Guaviare Tukanoan groups, and a second one formed by the Guayabero. 3. Tukanoan groups are found to be closer related to the Brazilian Amazonian populations than to the Guayabero.

**Conclusion:**

The results of this study suggest that the Guayabero group from Guaviare, are genetically differentiated from those Tukanoan groups of the Vaupés and Guaviare.

## Abstract (es)

**Introducción:**

En los departamentos del Vaupés y el Guaviare, al Suroriente de Colombia, en un área transicional entre la Amazonía y los Llanos Orientales, habitan pueblos indígenas de las familias lingüísticas Tucano (Oriental) y Guahibo. Se han hecho estudios sobre la cultura y la cosmología de dichos grupos, pero poco se ha investigado sobre su diversidad biológica y sus relaciones genéticas.

**Objetivo:**

Estimar la diversidad, la estructura y las relaciones genéticas de dos poblaciones Tucano Oriental y un grupo Guayabero (Guahibo) de la Amazonía colombiana.

**Metodos:**

Se recolectaron muestras de 106 individuos no emparentados, pertenecientes a comunidades indígenas del Vaupés y el Guaviare. Se tipificaron nueve microsatélites autosómicos. Se estimó la heterocigosidad, la diversidad génica, FST y FST pareados entre grupos. Se realizó una prueba de estructura espacial y se construyó un árbol de distancias genéticas con otras poblaciones indígenas amazónicas, Andinas y centroamericanas.

**Resultados:**

La diversidad genética se encuentra en el rango estándar de otras poblaciones amazónicas, pero es más baja que en población mestiza y afrodescendiente de Colombia. Los Tucano Oriental del Vaupés y el Guaviare conforman una unidad genética que se diferencia significativamente de los Guayaberos. Los grupos Tucano están más cerca genéticamente de las poblaciones amazónicas brasileras que de los Guayabero.

**Conclusión:**

Los resultados de este estudio sugieren que los Guayaberos en el Guaviare, están diferenciados genéticamente de los grupos Tucano del Vaupés y de los Tucano del Refugio en el Guaviare.

## Introduction

The Vaupés and Guaviare regions in southeastern Colombia are inhabited by several indigenous groups belonging to different linguistic families. These groups have an extensive history of demographic expansions, wars, alliances, migrations and trades. With the arrival of European populations into the region, a new chapter was opened, leading to the disruption of some cultural and social dynamics of the existing indigenous groups. To a large extent, it can be said that such disruptions caused a major reduction on the size of the indigenous populations, displacements from original territories, or even a total extinction of some groups^1^.

Currently, 24 ethnic groups live in this area representing about 30% of the national linguistic diversity. These groups have been classified^2^ into five linguistic families: Tukanoan (represented by the Eastern Tukanoan subfamily), Guahiban, Arawakan, Kakua-Nukak and Cariban. These ethnic groups are found scattered throughout both the Vaupés and Guaviare departments, East Tukanoan groups being the most widely distributed in the Vaupés, while in the Guaviare highlights predominates the presence of Guahibos, with some presence of Eastern Tukanoans whom in recent years have migrated from the Vaupés. Most of these ethnic groups live in indigenous communities with populations ranging from 22 to 6,222 inhabitants^3^ and some of them are currently in serious danger of disappearing, as is the case of the Eastern Tukanoan Pisamira in the Vaupés^2^.

Great efforts have been made towards an understanding of the social and cultural dynamics of the groups from this region^4^ ^-^ ^7^. However, very little (and very fragmented) information have been obtained at the genetic level^8^ ^,^ ^9^. With the aim to expand our biological knowledge on the populations of the Colombian Amazonia, nine autosomal microsatellites were typed to evaluate diversity, gene flow, genetic structure and degree of genetic differentiation between Eastern Tukanoan and Guahibo (Guayabero) native groups of the Vaupés and the Guaviare.

## Materials and Methods

### Samples.

**Figure 1:**
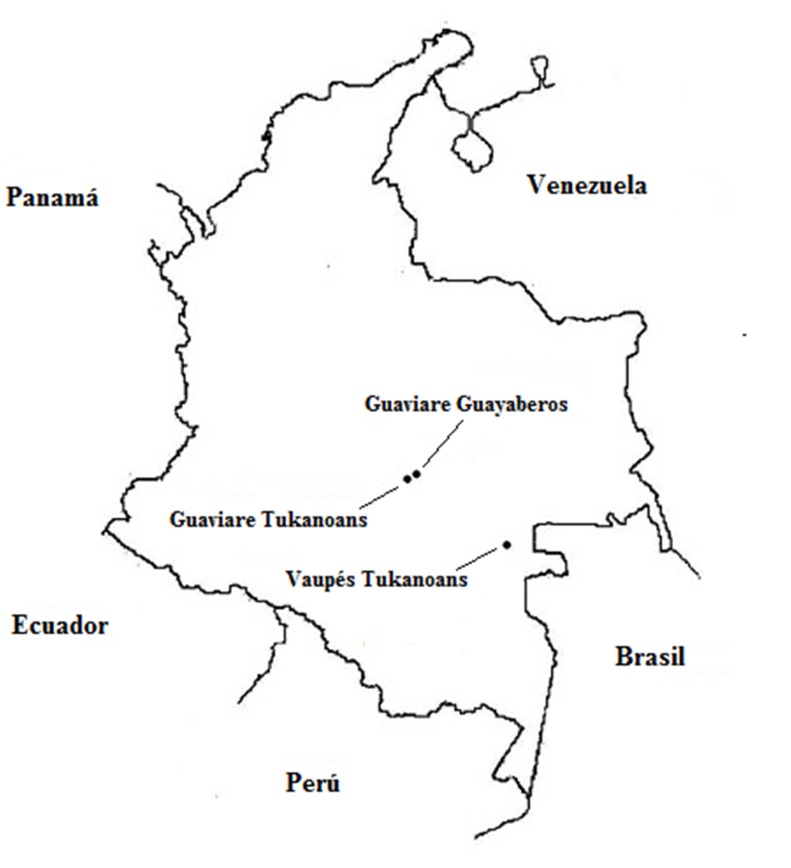
Map of continental Colombia with the location of the sampling sites: Mitú (Tucanos from Vaupés), El Refugio (Tucanos from Guaviare), and el Barrancón (Guayaberos from Guaviare)

For this study were collected biological samples of peripheral blood (4 mL) of 106 individuals belonging to indigenous groups in the departments of the Guaviare and the Vaupés, in southeastern Colombia (Fig. 1). The participants were healthy adults (men and women) without biological relationship (until the third degree of consanguinity). Samples were obtained with previous informed consent from each participant and with the agreement of the community leaders. This study was approved by the Institutional Human Ethics Review Committee at the Universidad del Valle, following the guidelines of the resolution 008430 of 1993 of the Ministry of Health of the Republic of Colombia.

### Populations.

The sample from Mitú (n= 55), named here Vaupés Tukanoans, groups Tukanoan^2^ speakers, some of whom live in the municipality of Mitú (1° 15'29 ''N, 70° 14'10'' O) and others are more like a ''floating population'' arriving in Mitú to trade goods and food but whose villages are elsewhere. The sample of El Refugio (2 ° 34'07 ''N, 72 ° 35'54'' W) in Guaviare (n= 22), named here Guaviare Tukanoans, groups Tukanoan speakers who migrated from the Vaupés about 50 years ago. The sample of El Barrancón (2° 15'29 ''N, 70° 14'10'' W) in the Guaviare (n= 29), named here Guaviare Guayaberos, groups people speaking Guayabero, classified within the Guahibo^2^ language family.

### Laboratory.

The DNA was extracted by the salting out procedure^10^. Each microsatellite (TH01, D7S820, D13S317, vWA, FGA, TPOX, F13B, LPL, FES / FPS) was amplified by PCR using primers whose sequences were obtained from STRBase^11^. The fragments were separated by polyacrylamide gel electrophoresis (8%), stained with silver nitrate. Each individual genotype was determined using allelic ladders constructed from DNA of previously established by capillary electrophoresis. 

### Data analysis.

The allele frequencies were obtained by counting; the intralocus independence (Hardy-Weinberg equilibrium) was tested. The observed heterozygosity (Ho) of each locus was calculated as the proportion of heterozygotes in each population. The observed heterozygosity of each population is the average heterozygosity across all loci. The gene diversity for each locus was calculated as n / (n-1) (1-Σp_i_^2)^, where n is the sample size and p_i_ is the frequency of allele i. The total gene diversity was calculated as the average diversity over all loci (assuming panmixia, genetic diversity is equivalent to the expected heterozygosity H*e*). The calculations were made with the software Arlequin 3.11^12^. 

Through an analysis of molecular variance, the degree of population genetic structure in two hierarchical levels was estimated: among groups (Vaupés Tukanoans, Guaviare Tukanoans and Guaviare Guayaberos) and among individuals within groups. This analysis divides the total variance into covariance components due to the intraindividual, interindividual and/or interpopulation differences. Covariance components were used to compute fixation indices, F*_ST_*. Gene flow was measured as Nem = (1/F*_ST _*-1)/4. The calculations were performed with Arlequin 3.11.

To determine the degree of differentiation among the three populations studied, exact test was performed based on the distribution of the genotypic frequencies, the calculations were made in GENEPOP version Web^13^. The Geneland^14^ software was used to implement a model of spatial and correlated allele frequencies and the number of populations K was estimated (with a maximum number of populations of 10 and 100,000 iterations), this software take into account the genotypic data and geographical position of the samples, regardless of linguistic affiliation.

The allelic frequencies of four loci (D7S820, D13S317, TH01, TPOX) were compared with data sets from six Amazonian populations, seven Andean and two Panamanian populations previously reported^15^ ^-^ ^18^. Genetic distances D*_A_* were calculated and graphically depicted with a Neighbor-joining tree (NJ) using the program POPTREE2^19^. The consistency was tested by bootstrap.

## Results

### Allelic frequencies.

The distribution of allelic frequencies of the Vaupés Tukanoans, Guaviare Tukanoans and Guaviare Guayaberos are shown in Table 1. The FGA was the microsatellite with the largest number of alleles and higher values of observed heterozygosity in the Guaviare Tukanoans and Guaviare Guayaberos samples (Table 2). The LPL was the system with the lowest number of alleles and observed heterozygosity in the Vaupés Tukanoans and the Guaviare Tukanoans. The FGA exhibited a deviation from the Hardy-Weinberg equilibrium in the Vaupés Tukanoans and Guaviare Guyaberos while systems D13S317, vWA, F13B, showed a significant deviation from HW equilibrium in Guaviare Tukanoans. Private alleles (relative to the three groups) were observed in the Vaupés Tukanoans at loci THO1 (allele 8), D13S317 (15), FGA (20), TPOX (9) and in the Guaviare Guayaberos at loci TPOX (7), F13B (6), FES/FPS (8). All these alleles have been previously reported for other Andean and Amazonian populations. Two individuals of the Vaupés Tukanoans presented TH01 allele 8, which had not been reported for this group.

**Table 1:**
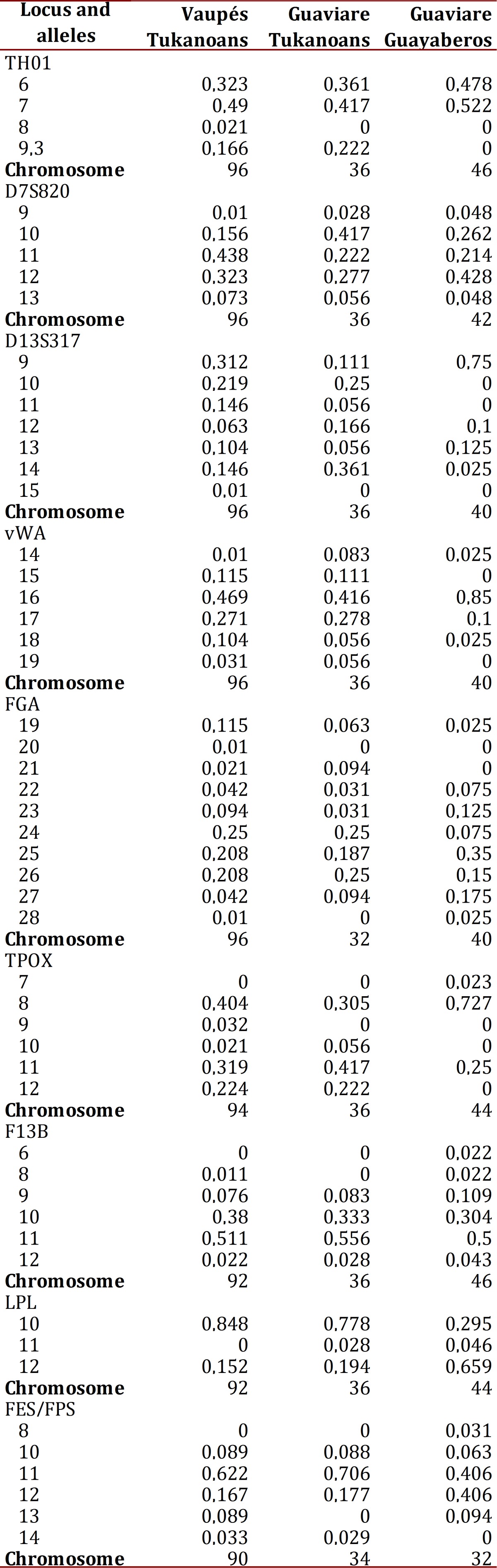
Allelic frequencies in nine autosomal STRs in three Amerindian populations of the Vaupés and the Guaviare departments, Southeast Colombia

### Genetic diversity.

The observed heterozygosity of Tukanoans and Guayaberos was found in the range 0.665-0.544, the gene diversity in the range 0.622-0.466 and the average number of alleles in the range 5.444-4.444 (Table 2). The Guaviare Guayaberos presented the lowest values of genetic diversity, although not significantly different from those found in Tukanoan groups.

**Table 2:**
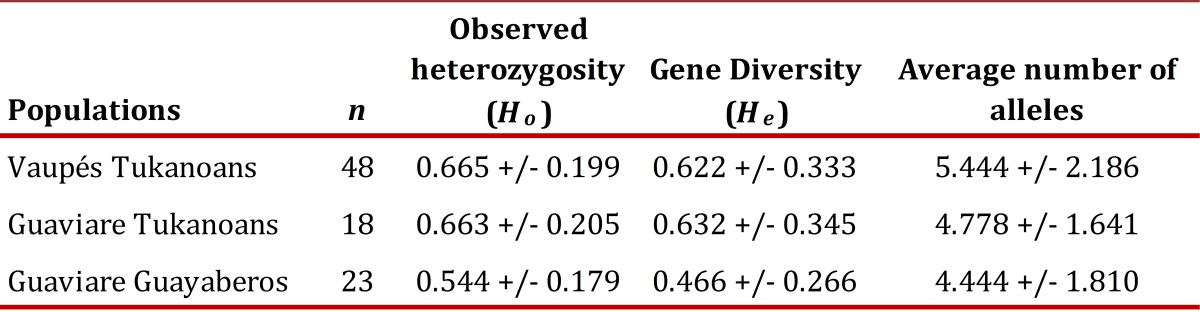
Genetic diversity indexes of three Amerindian populations of the Vaupés and the Guaviare.

### Genetic structure and population differentiation.

The F*_ST _*estimated by AMOVA for the three groups was 0.06691 (*p* <0.00001). The F*_ST_* estimated across all loci was 0.07477 (IC 95% 0.02897 - 0.13481). With respect to the pairwise F**_ST_** (Table 3), the Guayaberos showed moderate differentiation with high significance (*p* <0.01) regarding the two Tukanoans groups, while the FST between the two Tukanoan populations showed no significant differentiation (*p*> 0.05).

**Table 3:**
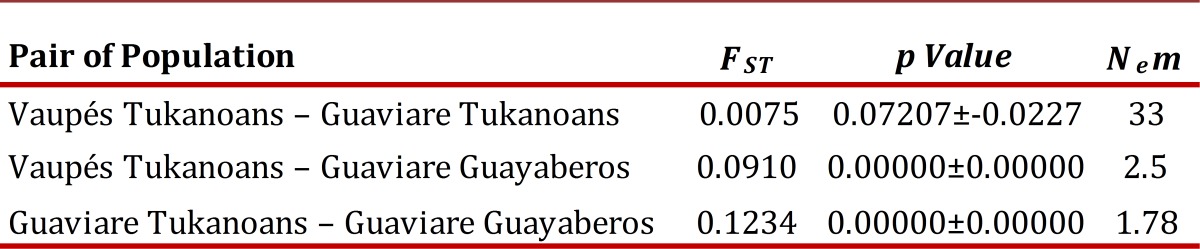
Pairwise F_ST_ values of the three Amerindian populations of the Vaupés and the Guaviare.

The exact test of population differentiation showed highly significant differences between Guayaberos and the two Tukanoans groups (*p* <0.01). However, it was not possible to detect any significant differences between the Vaupés Tukanoans and Guaviare Tukanoans, showing that genotypic frequencies of each locus have a similar distribution in the two populations. Similarly, the test of spatial differentiation in Geneland showed in all cases (ten separate runs to test the consistency of the results), the separation between Guayaberos and Eastern Tukanoan groups, with the two Tukanoan populations forming a single undifferentiated population.

N_e_m values show a low number of migrants between Guayaberos and the two populations of Eastern Tukanoan (Table 3), the opposite happens between the two Tukanoan populations showing a high rate of migration.

### Andes-Amazonia Genetic distances.

**Figure 2:**
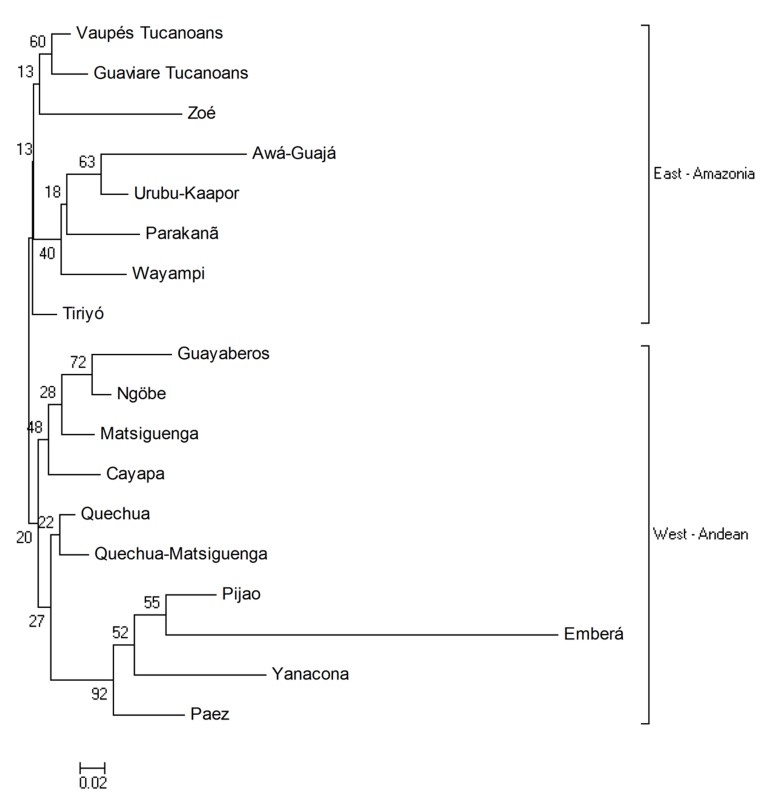
Neighbor-Joining tree based on D_A_ distances considering 16 South American Amerindian and 2 Panamanian populations (Emberá, Ngöbe). The numbers represent the bootstrap value. Present study: Vaupés Tukanoans, Guaviare Tukanoans, Guayaberos.

The NJ tree (Fig. 2) shows two clusters East-West: the Amazonian Tukanoan populations on a branch and Guayaberos, Andean and Panamanian groups on the other.

## Discussion

The genetic diversity of the Tukanoan populations (0.62 to 0.63) stands within the range reported for other Amerindian South American populations^16^ (0.554-0.753), which is lower than the African descendant or mestizo Colombian populations ^15^ ^,^ ^20^. These low diversity values compared to those of European and African human groups have been extensively reported ^21^ for other autosomal markers, Y chromosome, and mitochondrial DNA. Such low values could be related, first, with the peopling of America (by small populations that spanned Bering Strait to North America about 15,000-25,000 years ago, reaching South America around 15,000 years ago^22^, and second, with historical and demographic events that have caused the reduction in population sizes since the arrival of the Spaniards. The Guayaberos presented the lowest diversity indices, which although is not significantly different from the indices Tukanoans, might reflect a different biological history between these two groups. The presence of four private alleles in the Vaupés Tukanoans and three alleles in Guayabero can be interpreted as an additional evidence of the separation between these two groups. The lowest diversity of Guayaberos, with an expected heterozygosity (0.466) outside of the range found in other South American Amerindian populations (0.554-0.753), would be associated with historical events typical of nomadic and seminomadic groups, coupled with the loss of alleles caused by the reduction of their population sizes as a consequence of wars with other groups in the region in prehispanic times, in addition, some possible infectious diseases carried by the non-native people caused a massive amount of deaths among the Amerindian populations during the first half of last century ^23^. Also, more recently, the pressure of illegal armed groups have led to not only death of many indigenous peoples but also to the territorial displacement of such groups from their villages on the Ariari and Guayabero rivers to the current population of Barrancón^24^, destroying much of the cultural and genetic structure of these communities.

Additionally to the reduction of population sizes, another important factor in the structuring of the genetic variation in Guayaberos is the inbreeding marriage practices^25^. For these groups, kinship represents the major element of identity, and the marriages occur between bilateral cousins, a factor that contributes to the loss of genetic diversity. The Vaupés Tukanoans by contrast, associate to practices of linguistic^7^ exogamy, in which marriages are to occur between speakers of different language groups. In this exogamic marital-net, women migrate to the community of their future husbands, allowing a constant gene flow throughout the region. It is possible that this practice has helped to maintain average levels of diversity in populations with sizes not exceeding one thousand people, as in the vast majority of ethnic groups in the Vaupés^3^. However, a more detailed study with uniparental markers is needed to allow for a more in depth evaluation of the impact that matrilocality/ patrilocality oriented practices, asymmetry of gene flow, and inbreeding have had on the biological structure of the ethnic groups in the Vaupés region.

The Exact Test of population differentiation, the spatial structure test, and pairwise F*^ST^*, support the biological separation between the Guayaberos and Eastern Tukanoan groups. Culturally, Guayaberos have not participated, nor they currently do, in the exogamous system of the Vaupés. This lack of exogamous marital net is reflected in the results presented here. On the other hand, geographical proximity is not synonymous of a common biological history. The Guaviare Eastern Tukanoans are located near the Guayaberos (~ 7 km) ever since approximately five decades ago.

Nonetheless no evidence of marriages or a high gene flow between these communities were reported during the field work period carry out for this study. The estimated number of migrants between the Tukanoan and Guayabero groups was low compared to the number of migrants among the Vaupés Tukanoans and the Guaviare Tukanoans. This can potentially be a good evidence that in human populations socio-linguistic practices are crucial in the structuring of genetic variation; this can contribute or limit the gene flow between populations.

Comparison with other indigenous populations through the estimation of genetic distances group Tukanoans in the cluster of Amazonian populations. This grouping should not be interpreted as a common origin; nonetheless it could represent an ancient relationship among Tukanoans groups and other Brazilian Amazonian populations; these relationships are reflected in both oral tradition and cultural practices. The Tukanoan groups, for example, place their mythological origin in the ''milk lake'', an unspecified location downstream the Amazon River^6^. From there, an ancestral anaconda transported them (Tukanoans) upstream along the Vaupés river, and distributed the groups along the route. Such mythological story of origin is consistent with the genetic data analyzed here, which would support a possible origin of Tukanoan groups further southeast in the region now occupied by these groups in the Brazilian-Colombian border.

The case of Guayabero and Andean populations is similar to Tukanoan and the Amazonian populations. It is not possible to trace with certainty an origin of the Guayaberos, but the evidence here presented suggests that these populations may possibly have an Andean-Eastern plains origin more rather than an Amazon origin, notwithstanding some of the Guayabero's ancient practices- such as consumption of manioc-which presents some resemblances to the Tukanoan and Arawakan practices, in the Amazon Basin. The Guayabero location in ancient times (on the headwater of the Ariari and Guayabero rivers^25^, in the foothills of the Cordillera Oriental) and his nomadic condition, would have allowed them a greater mobility along the foothills and forests of the Orinoco savanna, thus presumably having more contact with Andean indigenous than with Amazonian groups.

Despite the genetic differentiation found between the Guayabero and Eastern Tukanoans groups, the interpretation of the East-West genetic relationships of these groups should take into account the limitations that carry the type and number of loci examined, sample sizes, and number of populations involved here. Interdisciplinary studies providing ethnographic, linguistic, and biological information of a higher number of Tukano, Guahibo, Arawak, Kakua-Nukak, and Puinave populations of the Eastern Plains and the Amazonia, are needed in order to establish a more reliable tree of the genetic relationships of these groups.

## Conclusions

1. The genetic diversity of Tukanoans is within the range of values of other Amazonian indigenous populations, which is lower than mestizo and Afro-descendant Colombian populations.

2. The test of population differentiation, the pairwise F_ST_ indices, and the test of spatial structure in geneland do not detect significant differences between the Vaupés Tukanoans and Guaviare Tukanoans.

3. The same tests detected significant differences between Eastern Tukanoans and Guayaberos, so it is concluded that although these two groups belonging to the Amazon region or transition to the eastern plains, have had an isolation level such today they constitute differentiated populations.

4. While there is a short distance between the community of El Refugio (Guaviare Tukanoans) and the community of El Barrancón (Guaviare Guayaberos), culture and language would be acting as a barrier to gene flow, maintaining a level of genetic differentiation between these two groups.

5. The Tukanoans are located closer to the populations of the Brazilian Amazon than the Andean populations. Opposite happens with the Guayabero, showing more proximity to the Andean populations than the Amazon.
